# Expression, prognostic value and mechanism of SP100 family in pancreatic adenocarcinoma

**DOI:** 10.18632/aging.204811

**Published:** 2023-06-22

**Authors:** Yunjie Duan, Yongxing Du, Yongrun Mu, Zongting Gu, Chengfeng Wang

**Affiliations:** 1 State Key Lab of Molecular Oncology and Department of Pancreatic and Gastric Surgery, National Cancer Center/National Clinical Research Center for Cancer/Cancer Hospital, Chinese Academy of Medical Sciences and Peking Union Medical College, Beijing, China; 2 Department of Hepatobiliary and Pancreatic Surgery and Minimally Invasive Surgery, Zhejiang Provincial People’s Hospital, Hangzhou Medical College, Hangzhou, Zhejiang, China; 3 Shanxi Province Cancer Hospital/Shanxi Hospital Affiliated to Cancer Hospital, Chinese Academy of Medical Sciences/Cancer Hospital Affiliated to Shanxi Medical University, Taiyuan, Shanxi, China

**Keywords:** pancreatic adenocarcinoma, SP100 family, TP53, M6A, biomarkers, prognosis, immune infiltration

## Abstract

Background: Pancreatic adenocarcinoma (PAAD) is one of the most aggressive malignancies with a very poor prognosis. Exploring more therapeutic targets and prognostic biomarkers is of great significance to improve the prognosis of PAAD patients. Increasing evidence supports that the speckled protein (SP) 100 family is associated with human cancer and immune disorders. However, the function of the SP100 family members in PAAD is still unclear.

Methods: R, Cytoscape, cBioPortal, and other software and online databases were used to comprehensively analyze the expression characteristics, prognostic value, and oncogenic mechanism of the SP100 family in PAAD.

Results: The high expression of SP100 family members in PAAD was significantly correlated with poor clinicopathological features and poor prognosis of PAAD patients. Mechanistically, TP53 mutations were significantly associated with the expression levels of the SP100 family members, which were significantly coexpressed with M6A methylation regulators and were activated in multiple oncogenic pathways, including the EMT pathways. Moreover, we found that their expression levels were significantly correlated with the sensitivity of multiple traditional chemotherapeutic drugs.

Conclusion: The SP100 family is closely related to the occurrence and development of PAAD and can be used as a new biomarker and therapeutic target for patients with PAAD.

## INTRODUCTION

PAAD stands as one of the most aggressive malignancies, ranking fourth among the leading causes of cancer-related mortality worldwide, accounting for approximately 4.5% of all cancer deaths [1]. Unfortunately, the diagnosis of PAAD often occurs at an advanced stage, hampering the efficacy of the most effective treatment option, early surgical resection. Despite significant advancements in identifying therapeutic targets ranging from genes, mRNAs, to miRNAs in the past decade [2], the clinical management of PAAD remains unsatisfactory. Hence, the exploration of additional therapeutic targets and prognostic biomarkers assumes critical importance in improving patient outcomes in PAAD.

The SP100 family encompasses four distinct members, namely SP100, SP110, SP140, and SP140L. Each member shares common features, characterized by the presence of HSR and SAND domains [3]. These highly similar proteins have been implicated in various human malignancies and immune disorders [4]. For instance, SP100 protein serves as a permanent resident of promyelocytic leukemia nuclear bodies and represents an autoantigenic target in primary biliary cirrhosis. The detection of anti-SP100 antibodies in serum aids in the diagnosis of this condition [5]. Notably, mutations in the SP110 gene have been identified as the causative factor in veno-occlusive disease with immunodeficiency, an autosomal recessive disorder characterized by severe combined T and B-cell immunodeficiency without the presence of lymphoid germinal centers [4]. Additionally, polymorphisms in the SP140 gene have been linked to an increased risk of multiple sclerosis. The loss of SP140, resulting from genetic variants, disrupts effective innate immunity, thereby destabilizing the intestinal environment [3]. Furthermore, the SP140L protein localizes to nuclear bodies and represents a novel autoantigen observed in patients with primary biliary cirrhosis [5].

In this study, we conducted a comprehensive investigation into the potential functions and underlying mechanisms of SP100 family members in the development and progression of PAAD by leveraging public databases and employing multiple bioinformatics analysis techniques. Our research commenced by elucidating the differential expression patterns of SP100 family members across various cancer types, followed by an in-depth analysis of the correlation between their expression levels and the prognosis of PAAD patients. Furthermore, we proposed plausible mechanisms through which SP100 family members contribute to the initiation and advancement of PAAD, incorporating perspectives such as gene variation, gene enrichment, gene coexpression, protein interactions, and drug sensitivity. Collectively, our findings unveil the oncogenic role played by SP100 family members in PAAD, highlighting their potential as promising therapeutic targets and prognostic biomarkers for PAAD patients.

## MATERIALS AND METHODS

### Analysis of expression

TCGA is a large-scale cancer genomics research project that encompasses samples from over 30 types of cancer. TCGA-PAAD, for instance, comprises samples from 176 patients with PAAD, including tumor and normal control tissues. The tumor tissues encompass primary and metastatic lesions, while the normal control tissues include healthy pancreatic and related tissues. Multiple types of data analyses are available, including genomic variations, gene expression profiles, and protein expression profiles [6]. The expression levels of SP100 family members in cancer tissues, adjacent noncancerous tissues, and normal pancreatic tissues were analyzed using R (version 3.6.3) based on data from the PAAD project of The Cancer Genome Atlas (TCGA) database and the pancreas project of the Genotype-Tissue Expression (GTEx) database. Before conducting analyses on TCGA and GTEx data using the R package, we performed a uniform data transformation on the extracted TCGA and GTEx data using log2 (value+1). We then used the prcomp() function from the stats package to detect batch effects in the data and used the ComBat() function from the sva package to correct for these batch effects. We then analyzed the expression signatures of SP100 family members at the single-cell level using R (version 3.6.3) based on a previously published PAAD single-cell dataset (GSE154778). GSE154778 is a single-cell RNA sequencing dataset that can be used to study the heterogeneity and tumor microenvironment of pancreatic cancer. The dataset comprises high-quality transcriptome data from a total of 8000 cells from 10 primary tumors and 6926 cells from 6 metastatic samples. The number of cells obtained from each patient ranges from 143 to 1570 primary tumor cells and 125 to 2885 biopsy cells from metastases [7].

### Analysis of clinicopathological features and prognosis

Based on data from the PAAD project of the TCGA database (Supplementary Table 1), the relationship between the expression levels of SP100 family members and patient clinicopathological characteristics was analyzed using R (version 3.6.3). The Kaplan-Meier Plotter database (http://www.kmplot.com/) is an online survival analysis tool that enables univariate and multivariate survival analysis using any customized data [8]. We utilized this tool to analyze the correlation between the expression levels of SP100 family members and PAAD patient overall survival (OS) and recurrence-free survival (RFS). Analysis and plotting of receiver operating characteristic (ROC) curves were performed using the “pROC” R package (version 1.17.0.1) and the “ggplot2” R package (version 3.3.3). The area under the curve (AUC) was between 0.5 and 1.

### Analysis of gene variation and carcinogenic mechanisms

The cBioPortal database (http://www.cbioportal.org/) is a web resource for exploring, visualizing, and analyzing multidimensional cancer genomic data [9]. We analyzed the gene variant profiles of SP100 family members in PAAD and their relationships with some clinicopathological features using the cBioPortal database. The TIMER database (http://timer.cistrome.org/) provides six main analysis modules, allowing the user to interactively explore the link between immune infiltration and a series of factors, including gene expression, clinical, and somatic mutation and somatic copy number variation data [10]. Based on this database, we analyzed the correlation between the expression of members of the SP100 family and the expression of M6A methylation regulators and the correlation between the expression of members of the SP100 family. The UALCAN database (http://ualcan.path.uab.edu) is an easy-to-use interactive portal enabling in-depth analysis of TCGA gene expression data [11]. We used this database to analyze the correlation between the expression level and promoter methylation level of SP100 family members and TP53 mutation status. The LinkedOmics database (http://www.linkedomics.org/) contains 32 kinds of multi-omics data and clinical data of cancer and is the first integrated database of clinical proteomics tumor of global protein information based on mass spectrometry analysis of multiomics data [12]. We used this database to identify the 400 genes that were most closely related to the total expression of SP100 family members. The Metascape database (https://metascape.org) is a web-based portal designed to provide experimental biologists with a comprehensive resource for annotating and analyzing gene lists [13]. We utilized this database to visualize the biological process (BP), cellular component (CC), molecular function (MF), and Kyoto Encyclopedia of Genes and Genomes (KEGG) terms of SP100 family members and their 400 coexpressed genes. The GSCALite database (http://bioinfo.life.hust.edu.cn/web/GSCALite/) can be used to dynamically analyze and visualize gene sets related to the drug sensitivity of cancers and has broad practical value for cancer researchers [14]. We used this database to conduct a carcinogenic pathway enrichment analysis of members of the SP100 family. The STRING database (https://string-db.org/) and Cytoscape (version 3.9.1) were used to construct a functional network comprising the genes with the strongest protein–protein interactions (PPIs) with SP100 family members and to score the strength of association of these genes [15, 16].

### Immune infiltration and drug sensitivity analyses

TISIDB (http://cis.hku.hk/TISIDB/) integrates multiple types of tumor immunology data resources, and by using this database, researchers can cross validate the role of genes of interest in tumor immune interactions based on literature mining and high-throughput data analysis and generate testable hypotheses and high-quality data for publication [17]. We used this database to analyze the correlations of the expression levels of SP100 family members with the levels of tumor-infiltrating immune cells and the expression levels of immune molecules in PAAD. The TIMER database was used to analyze the correlation between the change in the copy number of SP100 family members and the infiltration levels of six kinds of immune cells in PAAD. In addition, TISIDB was also used to analyze differences in the expression levels of SP100 family members in different immune subtypes of PAAD. Next, R (version 3.6.3) was used to analyze the correlation between the expression of SP100 family members and the expression of multiple immune checkpoint genes. Finally, the GSCALite database was used to analyze the relationship between the expression levels of SP100 family members and sensitivity to multiple chemotherapy or targeted drugs.

### Experimental validation of bioinformatic data

We used the SP100 antibody (Ag1939) from Proteintech to perform immunohistochemical staining on tumor and adjacent tissues from 92 PAAD patients and the score of expression level is equal to staining intensity multiplied by positive rate [18]. We then analyzed the correlation between SP100 levels and clinical-pathological characteristics of the patients using R packages such as ggplot2, ggalluvial, and survival. Additionally, we performed Cox regression analysis using R packages such as surviva and rms. R version 3.6.3 was used for all analyses.

### Data availability

The original contributions presented in the study are included in the article Supplementary Material, further inquiries can be directed to the corresponding author.

## RESULTS

### Members of the SP100 family are highly expressed in some cancer tissues

**Figure 1:**
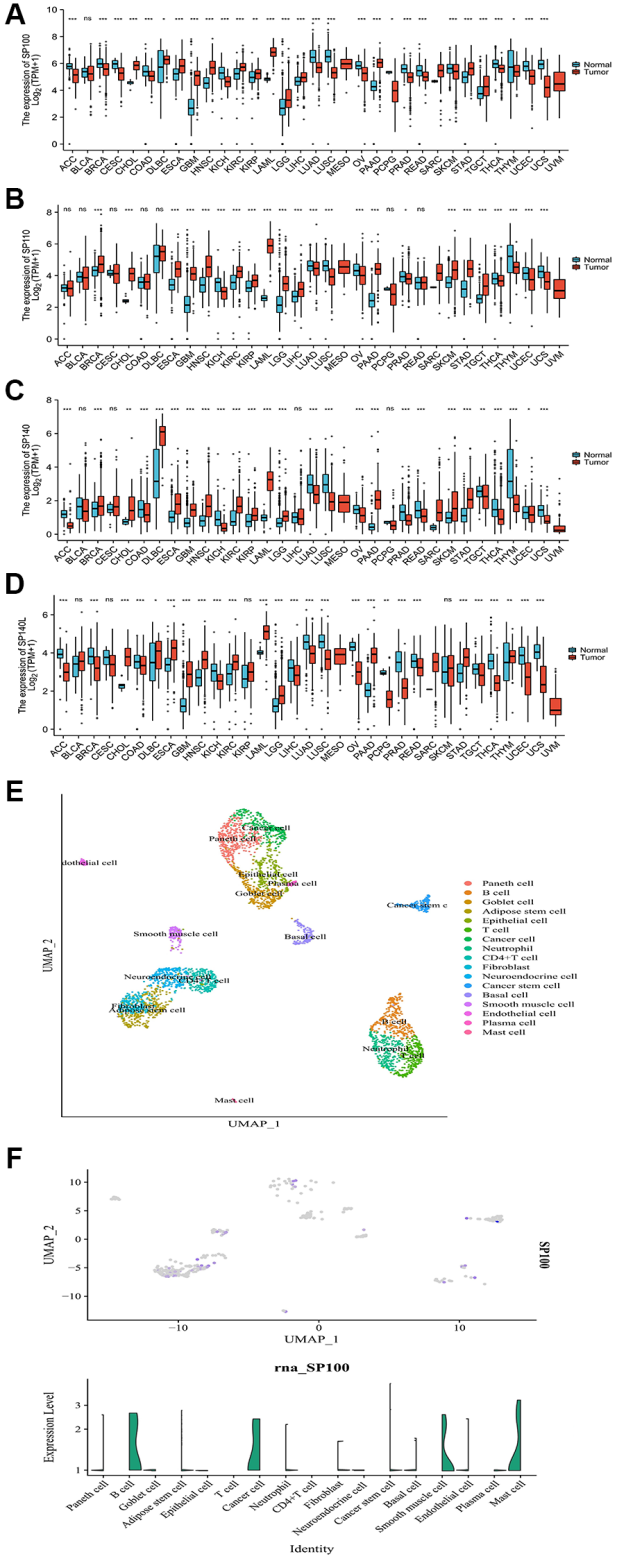
**Expression characteristics of SP100 family at the mRNA level in pan-cancer and the single-cell level in PAAD.** (**A**) The mRNA level of SP100; (**B**) The mRNA level of SP110; (**C**) The mRNA level of SP140; (**D**) The mRNA level of SP140L. ns, *p* ≥ 0.05; ^*^p < 0.05; ^**^p < 0.01; ^***^p < 0.001; (**E**) The cell group; (**F**) The single-cell level of SP100.

By leveraging data from the PAAD project of TCGA database and the Pancreas project of the Genotype-Tissue Expression (GTEx) database, we have unveiled the distinct mRNA expression profiles of SP100 family members in multiple cancer tissues compared to adjacent paracancerous and normal tissues. Notably, mRNA expression levels of SP100 family members were consistently elevated in nine cancer tissues, including PAAD, cholangiocarcinoma (CHOL), and stomach adenocarcinoma (STAD), when compared to their respective noncancerous counterparts. Conversely, the mRNA expression levels in eight other cancer tissues, such as lung adenocarcinoma (LUAD), ovarian serous cystadenocarcinoma (OV), and uterine corpus endometrial carcinoma (UCEC), exhibited lower levels compared to noncancerous tissues (Figure 1A–1D). Furthermore, employing single-cell datasets specifically focusing on PAAD, our analysis revealed that SP100 family members exhibited higher expression levels in cancer cells and cancer stem cells, while demonstrating lower expression levels in various tumor-infiltrating immune cells, including T cells and neutrophils (Figure 1E, 1F; Supplementary Figure 1). These findings collectively suggest that SP100 family members may fulfill crucial roles in the initiation and progression of PAAD, as indicated by their significantly heightened expression levels in PAAD tissues when contrasted with corresponding noncancerous tissues.

### High expression of SP100 family members in PAAD is associated with poorer clinicopathological features and prognosis of patients

**Figure 2:**
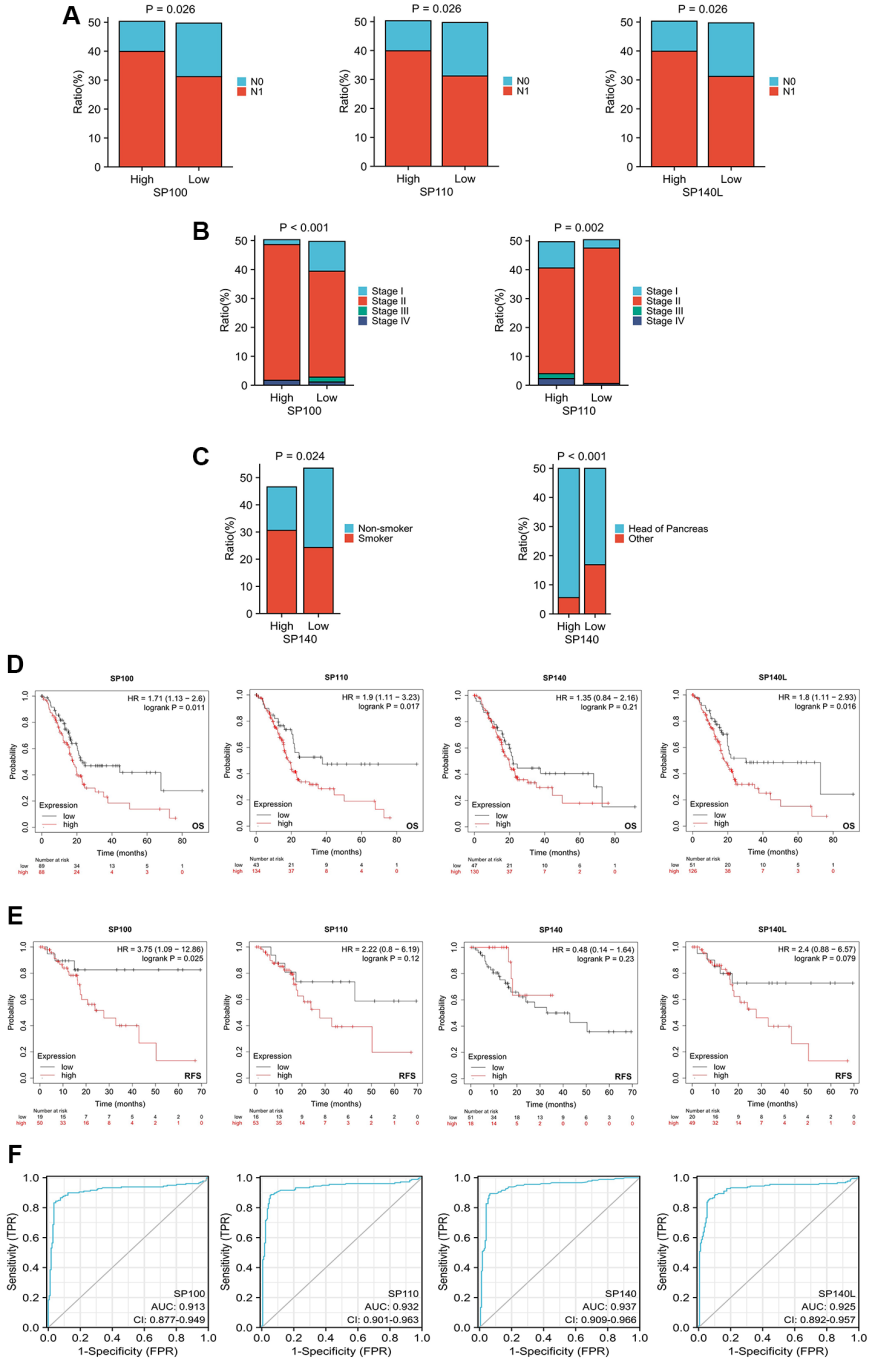
**Relationship between expression levels of SP100 family members and clinicopathological features and prognosis of patients with PAAD.** (**A**) The proportion of patients with N1 stage in PAAD patients with high SP100/SP110/SP140L expression was higher; (**B**) The proportion of patients with Stage IV stage in PAAD patients with high SP100/SP110 expression was higher; (**C**) The proportion of patients with smoking history and tumors located in the pancreatic head in PAAD patients with high SP140 expression was higher; (**D**) The increased expression level of SP100/SP110/SP140L were significantly correlated with shorter OS; (**E**) The increased expression level of SP100 were significantly correlated with shorter RFS; (**F**) SP100 family members showed high accuracy in predicting normal and neoplastic outcomes.

Our comprehensive investigation into the correlation between the expression levels of SP100 family members and the clinicopathological features and prognosis of patients with PAAD has unraveled the influential role played by these members in the initiation and progression of PAAD. Notably, our findings highlight intriguing associations between the expression levels of SP100, SP110, SP140L, and SP140, and various clinicopathological features. For instance, a higher proportion of PAAD patients exhibiting elevated expression of SP100, SP110, or SP140L were observed to have N1 stage disease, indicating a potential connection between increased expression of these family members and disease progression (Figure 2A; Supplementary Table 2). Furthermore, higher expression levels of SP100 and SP110 were found to be associated with stage IV disease, further implicating their involvement in advanced stages of PAAD (Figure 2B; Supplementary Table 2). Additionally, a higher proportion of PAAD patients with elevated SP140 expression had a history of smoking and tumors located in the head of the pancreas, suggesting potential links between SP140 expression, smoking behavior, and tumor localization (Figure 2C; Supplementary Table 2). Importantly, our investigation also shed light on the prognostic significance of SP100 family members. The expression levels of SP100, SP110, and SP140L were significantly associated with overall survival (OS) (Figure 2D), whereas SP100 expression exhibited a significant association with recurrence-free survival (RFS) (Figure 2E). These findings imply that elevated expression levels of SP100 family members may serve as valuable prognostic indicators for patients with PAAD. Furthermore, our analysis demonstrated that the expression levels of SP100 family members exhibited robust discrimination between normal pancreatic tissue and PAAD tissue, as evidenced by high Area Under the Curve (AUC) values in Receiver Operating Characteristic (ROC) analysis (Figure 2F). This suggests that SP100 family members possess exceptional accuracy in distinguishing between normal and tumor tissues, further emphasizing their potential utility as reliable diagnostic markers for PAAD. In summary, our comprehensive investigation has revealed compelling associations between the expression levels of SP100 family members and clinicopathological features and prognosis in PAAD patients. These findings underscore the crucial role played by SP100 family members in PAAD development and progression and further support their potential as promising therapeutic targets and diagnostic biomarkers.

### Gene variation characteristics of SP100 family members in PAAD and their relationship with M6A methylation regulator expression and TP53 mutation status

**Figure 3:**
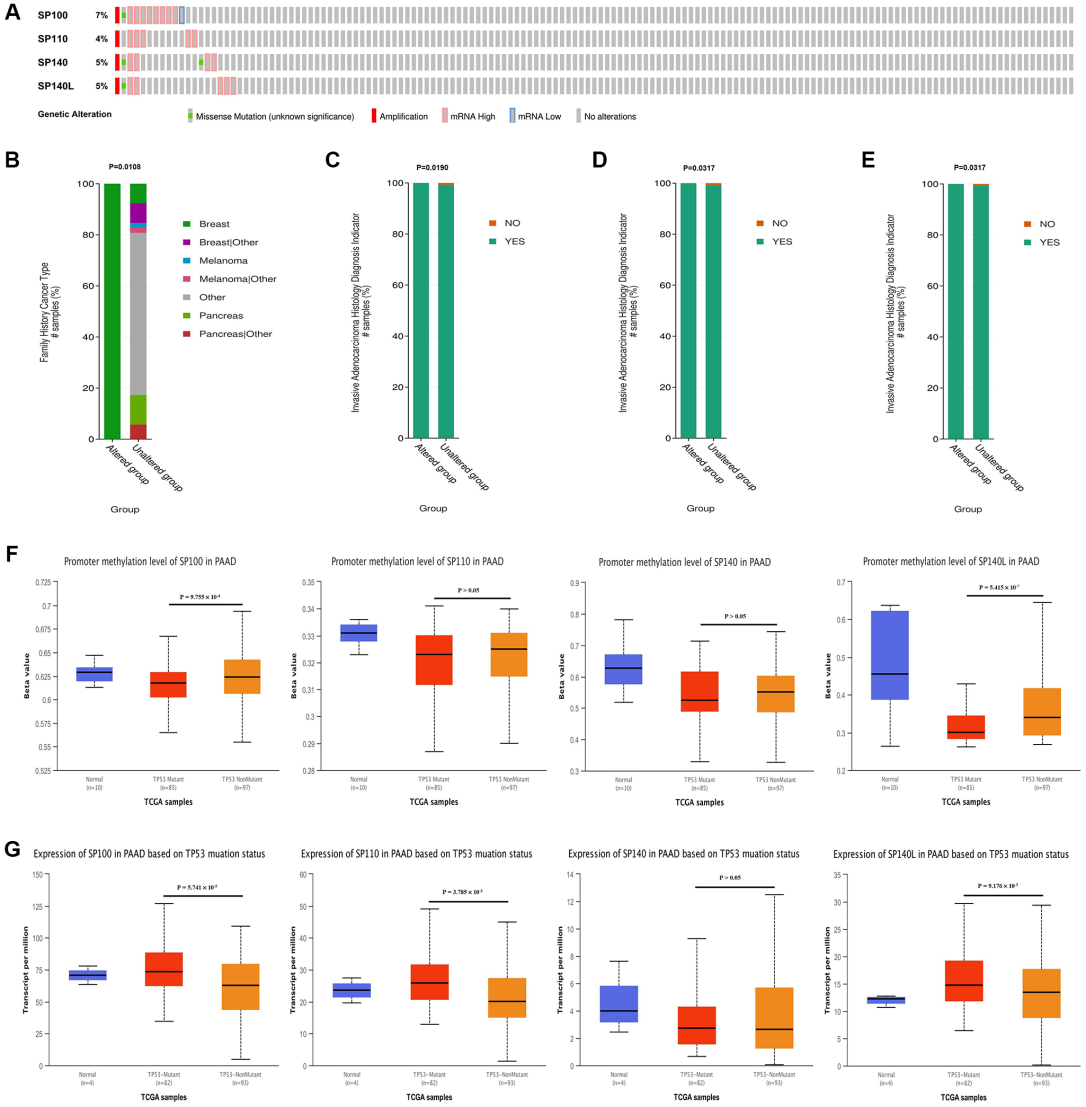
**Genetic variation characteristics of SP100 family members in PAAD and relationship of their expression levels with m6A methylation regulators and TP53 mutation status.** (**A**) Genetic variation characteristics of the SP100 family members in PAAD; (**B**) The amplification and mRNA High mutation of SP100 was significantly correlated with PAAD patients have a family history of breast cancer; (**C**–**E**) The amplification and mRNA High mutation of SP110/SP140/SP140L was significantly correlated with PAAD invasion of surrounding tissues; (**F**) The promoter methylation level of SP100/SP140L in the TP53 mutant PAAD tissue was significantly lower than that in the TP53 wild-type PAAD tissue; (**G**) The expression level of SP100/SP110/SP140L in the TP53 mutant PAAD tissue was significantly higher than that in the TP53 wild-type PAAD tissue.

Utilizing data from the cBioPortal database, we conducted a comprehensive analysis of the genetic variations exhibited by members of the SP100 family in PAAD. Intriguingly, gene mutations were observed in 19 samples from PAAD patients, accounting for approximately 13% of the cases analyzed. These mutations primarily manifested as high amplifications and mRNA-related alterations. Notably, among the SP100 family members, SP100 exhibited the highest mutation frequency, affecting approximately 7% of the cases (Figure 3A). Furthermore, our analysis uncovered potential associations between SP100 family mutations and specific clinical characteristics of PAAD patients. Mutation of SP100 appeared to be linked to a family history of breast cancer in PAAD patients (Figure 3B). Moreover, mutations in SP110, SP140, and SP140L were potentially associated with more aggressive PAAD tumors (Figure 3C–3E). Another significant finding of our study was the positive correlation between the expression levels of SP100 family members and the vast majority of M6A methylation regulators (Supplementary Figure 2). This observation suggests a potential interplay between the expression of SP100 family members and M6A methylation regulators, thereby implicating their collective involvement in PAAD pathogenesis. Additionally, our investigation delved into the relationship between TP53 mutations and SP100 family members. Notably, in comparison to wild-type TP53 PAAD tissue, TP53-mutant PAAD tissue exhibited elevated expression levels of SP100 family members, with statistically significant differences observed for SP100, SP110, and SP140L (Figure 3G). Furthermore, TP53 mutation was associated with reduced promoter methylation levels of SP100 family members, with statistically significant differences observed for SP100 and SP140L (Figure 3F). These findings suggest that TP53 mutations may contribute to increased expression levels of SP100 family members, indicating a potential regulatory role of TP53 in modulating these members. Moreover, considering the coexpression of SP100 family members with numerous M6A methylation regulators, it is plausible that increased expression of SP100 family members may further enhance the expression levels of M6A methylation regulators. The intricate interplay among these factors may underlie the mechanistic aspects through which SP100 family members promote the development of PAAD. Overall, our comprehensive analysis of genetic variations in SP100 family members in PAAD reveals a predominance of amplifications within this gene family. Additionally, our findings suggest a potential regulatory relationship between TP53 mutations, increased expression of SP100 family members, and the expression of M6A methylation regulators. These insights shed light on the complex mechanisms underlying the role of SP100 family members in driving PAAD development.

### Gene enrichment analysis of SP100 family members and their 400 coexpressed genes

**Figure 4:**
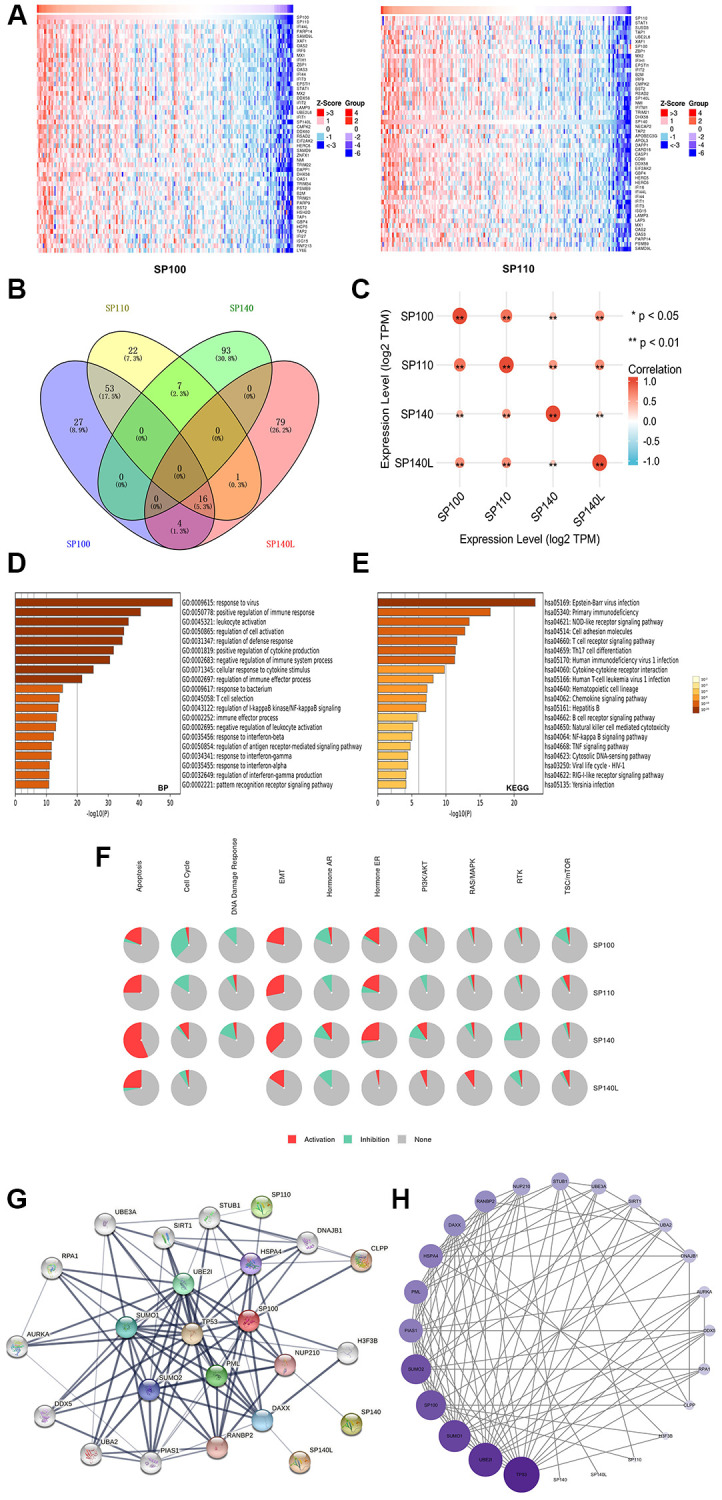
**Gene enrichment analysis and PPI network construction of SP100 family in PAAD.** (**A**) Top 50 genes co-expressed with SP100/SP110; (**B**) The Venn diagram of SP100 family members and their 400 co-expressed genes; (**C**) SP100 family members are also co-expressed among themselves. ^*^p < 0.05; ^**^p < 0.01; (**D**) The GO enrichment of the BP terms of the SP100 family and its 400 co-expressed genes; (**E**) The KEGG enrichment of the SP100 family and its 400 co-expressed genes; (**F**) SP100 family played an activating role in a variety of oncogenic pathways; (**G**, **H**) TP53 played an important role in PPI network which was closely related to SP100 family.

Using the LinkedOmics database, we conducted an in-depth analysis to identify the 400 genes exhibiting the strongest coexpression relationships with SP100 family members. The heatmap displayed the top 200 genes among them, offering valuable insights into their coexpression patterns (Figure 4A) (Supplementary Figure 3) (Supplementary Table 3). Moreover, utilizing the Wayne map, we identified 16 genes (SP100, SP110, XAF1, IRF9, SP140L, SAMD9, NMI, DAPP1, PSMB9, TRIM21, HSH2D, TAP1, HCP5, RTP4, HLA-F, and IRF1) that exhibited robust coexpression with SP100/SP110/SP140L (Figure 4B) (Supplementary Table 4). Notably, there was a significant level of coexpression among the members of the SP100 family itself (Figure 4C) (Supplementary Table 5). Subsequently, leveraging the Metascape database, we performed GO and KEGG enrichment analyses on both the SP100 family members and their 400 coexpressed genes (Supplementary Table 6). The GO analysis highlighted their prominent enrichment in biological processes (BP) such as “response to virus,” “positive regulation of immune response,” “leukocyte activation,” “side of membrane,” and "immunological synapse" (Figure 4D) (Supplementary Figure 4A). Furthermore, the cellular component (CC) terms demonstrated enrichment in “plasma membrane protein complex,” “double-stranded RNA binding,” and “kinase binding” (Supplementary Figure 4A). Additionally, the molecular function (MF) terms indicated involvement in “immune receptor activity” (Supplementary Figure 4B). The KEGG analysis revealed significant enrichment in terms such as “primary immunodeficiency,” “NOD-like receptor signaling pathway,” and “cell adhesion molecule-related pathway” (Figure 4E). Building upon these findings, we utilized the GSCALite database to further explore the roles of SP100 family members in various carcinogenic pathways. Notably, the analysis revealed their significant contributions to the epithelial-mesenchymal transition (EMT) and hormone/estrogen receptor (ER) pathways, indicating their substantial involvement in promoting PAAD (Figure 4F). Moreover, SP140L was found to be implicated in the PI3K/AKT and RAS/MAPK pathways, which are known to facilitate tumor cell growth and proliferation [19, 20]. These observations suggest that the activation of SP100 family members in diverse carcinogenic pathways may serve as a mechanistic basis underlying the promotion of PAAD occurrence and development. These comprehensive analyses shed light on the coexpression patterns, functional enrichment, and pathway involvement of the SP100 family members, revealing their significant roles in PAAD pathogenesis.

### Construction and analysis of a PPI network for the SP100 family members

To investigate the protein-protein interaction (PPI) network involving the SP100 family members in PAAD, we employed the STRING database to identify the 24 genes with the strongest PPIs with the SP100 family members (Supplementary Table 7). Subsequently, Cytoscape software (version 3.9.1) was utilized to map and analyze the corresponding PPI networks. In the visual representation, larger circles and darker colors indicated a higher number of PPIs associated with a specific gene. Notably, within the SP100 family member PPI network, TP53 emerged as a central player (Figure 4G), exerting a substantial influence on other genes (Figure 4H) (Supplementary Table 8). This observation suggests that TP53 likely holds a pivotal role in the PPI network, potentially regulating the SP100 family members. These findings indicate a potential regulatory relationship between TP53 and the members of the SP100 family, unveiling a noteworthy aspect of their interplay in PAAD.

### Relationships of SP100 family members with immune factors in PAAD

**Figure 5:**
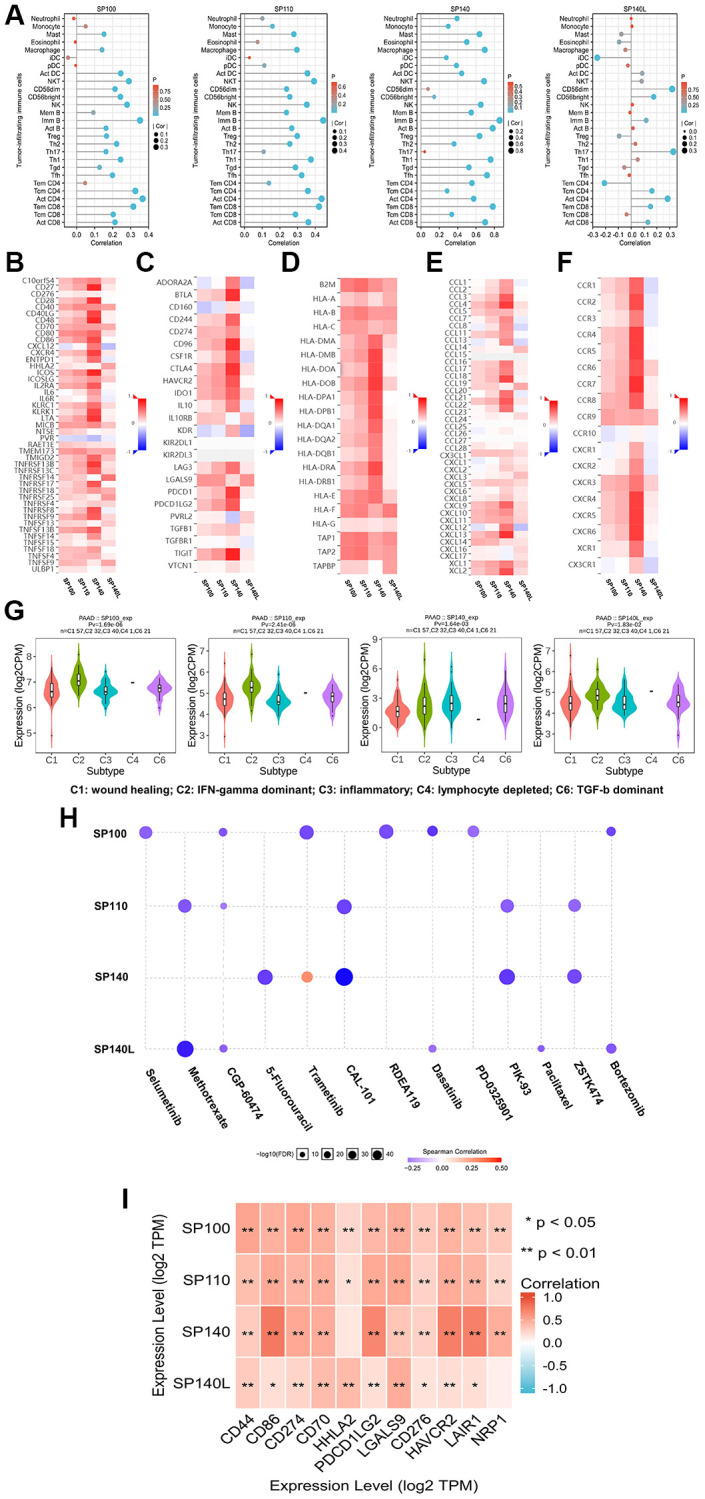
**The immune landscape of SP100 family in PAAD.** (**A**) The expression level of SP100/SP110/SP140 was positively correlated with the infiltration level of a variety of tumor infiltrating immune cells, including Act B, Tcm CD8 and Tem CD8, but SP140L was the opposite; (**B**–**F**) The expression level of SP100/SP110/SP140 was positively correlated with most immunomodulators, but SP140L was the opposite; (**G**) The expression of SP100 family was significantly different among the five immune subtypes; (**H**) The expression of SP100 family was positively correlated with the sensitivity of various targeting or chemotherapeutic drugs. (**I**) There are significant co-expression correlations between SP100 family members and several immune checkpoint genes, including CD44, HHLA2 and HAVCR2. ^*^p < 0.05; ^**^p < 0.01.

Using the TISIDB database, we conducted an analysis to explore the relationship between the expression of the SP100 family members and the levels of tumor-infiltrating immune cells, as well as multiple immunomodulators (Figure 5A–5F). The findings revealed positive correlations between the expression levels of SP100/SP110/SP140 and most tumor-infiltrating immune cells, including activated B cells, Tcm cells, CD8 T cells, and NKT cells (Supplementary Table 9). Moreover, the expression levels of SP100/SP110/SP140 displayed positive associations with most immunomodulators, encompassing immune promoters, MHC molecules, chemokines, and chemokine receptors (Figure 5B, 5D–5F). Notably, SP140L exhibited a neutral regulatory effect on PAAD immunity (Figure 5A, 5B, 5D–5F) (Supplementary Table 9). Another intriguing observation was the significant differences in the expression levels of SP100 family members across different immune subtypes of PAAD: SP100/SP110/SP140L displayed the highest expression in C2 subtype PAAD tissues, while the lowest expression was observed in C3 subtype PAAD tissues (Figure 5G). Conversely, SP140 exhibited the highest expression in C3 subtype PAAD tissues and the lowest expression in C1 subtype PAAD tissues (Figure 5G). Collectively, these findings illustrate the complex and diverse roles of SP100 family members in immunoregulation within the PAAD tumor microenvironment.

### The potential value of SP100 family members in clinical treatment

Utilizing the GSCALite database, we conducted an analysis to explore the associations between the expression levels of the SP100 family members and sensitivity to various chemotherapy and targeted agents. Intriguingly, the results revealed a positive correlation between the expression levels of SP140 and sensitivity to 5-fluorouracil, while SP140L expression was positively correlated with sensitivity to paclitaxel (Figure 5H). Therefore, PAAD patients with elevated expression of SP140 and SP140L may exhibit improved response to these specific drugs. Additionally, leveraging data from the TIMER database, we examined the relationship between the expression levels of the SP100 family members and multiple cancer-promoting immune checkpoint genes. The findings demonstrated significant coexpression between members of the SP100 family and various immune checkpoint genes, including CD44 and HAVCR2 (Figure 5I) (Supplementary Table 10). This observation holds significant implications for the treatment of PAAD, as the development of immune checkpoint inhibitors targeting these genes represents a promising approach. In conclusion, the members of the SP100 family hold potential as biomarkers for predicting sensitivity to PAAD treatment. Further experimental studies and the development of drugs targeting the SP100 family members are urgently warranted, and their outcomes are highly anticipated.

### High expression of SP100 is an independent risk factor for poor prognosis in PAAD patients

**Figure 6:**
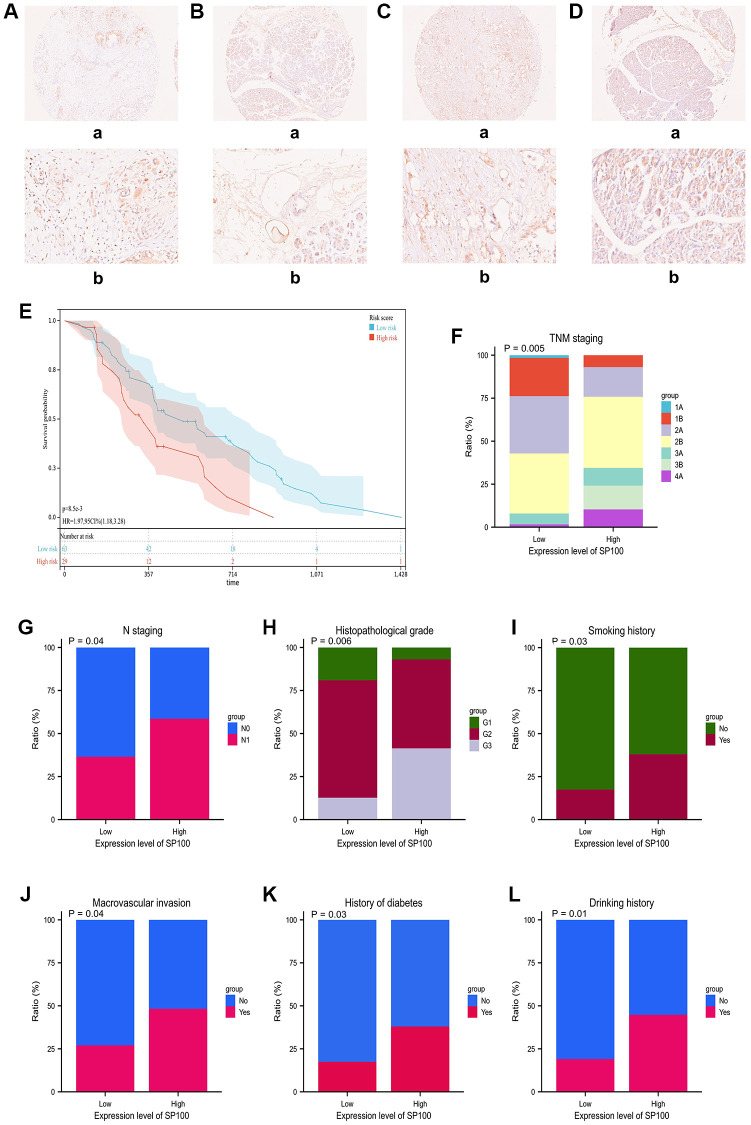
**The immunohistochemical staining characteristics and expression levels of SP100 in PAAD patient tissues and their correlation with clinical features of PAAD patients.** (**A**, **B**) The immunohistochemical staining features of SP100 in the pancreatic cancer tissue and adjacent tissue of patient ZCPA190705 were examined at 10X (**a**) and 40X (**b**) magnification, respectively; (**C**, **D**) The immunohistochemical staining features of SP100 in the pancreatic cancer tissue and adjacent tissue of patient ZCPA190713 were examined at 10X (**a**) and 40X (**b**) magnification, respectively; (**E**) The increased expression level of SP100 was significantly correlated with shorter OS; (**F**) The increased expression level of SP100 was significantly correlated with later TNM staging; (**G**) The increased expression level of SP100 was significantly correlated with later N staging; (**H**) The increased expression level of SP100 was significantly correlated with higher pathological grade; (**I**) The increased expression level of SP100 was significantly correlated with smoking history; (**J**) The increased expression level of SP100 was significantly correlated with macrovascular invasion; (**K**) The increased expression level of SP100 was significantly correlated with history of diabetes; (**L**) The increased expression level of SP100 was significantly correlated with drinking history.

We conducted immunohistochemical staining on the cancerous and adjacent tissues of 92 patients with PAAD using the SP100 antibody (Ag1939) from Proteintech Company. Our results showed that the expression levels of SP100 were significantly higher in cancerous tissues than in adjacent tissues. Here, we present the staining results of two patients’ cancerous (Figure 6A, 6C) and adjacent tissues (Figure 6B, 6D). Furthermore, we analyzed the clinical characteristics of these 92 PAAD patients (Supplementary Table 11) and found that those with high expression levels of SP100 had significantly shorter overall survival time (*p* = 0.006) (Figure 6E). Additionally, high SP100 expression was positively correlated with later TNM stage (*p* = 0.005) (Figure 6F), later N stage (*p* = 0.04) (Figure 6G), higher tumor pathological grade (*p* = 0.006) (Figure 6H), smoking history (*p* = 0.03) (Figure 6I), tumor invasion of major blood vessels (*p* = 0.04) (Figure 6J), diabetes history (*p* = 0.03) (Figure 6K), and alcohol consumption history (*p* = 0.01) (Figure 6L). Furthermore, Cox regression analysis showed that the expression level of SP100 was a risk factor and an independent prognostic factor (Table 1). These findings indicate that the expression level of SP100 is significantly higher in cancerous tissues of PAAD patients compared to adjacent tissues, and high expression of SP100 is significantly associated with poor prognosis in PAAD patients.

**Table 1:** The results of COX regression analysis for prognostic risk factors in 92 patients with PAAD.

**Characteristics**	**Total (*N*)**	**Univariate analysis**		**Multivariate analysis**	
		**Hazard ratio (95% CI)**	***P* value**	**Hazard ratio (95% CI)**	***P* value**
Age (year)	92	0.991 (0.966–1.018)	0.52		
Sex	92		0.271		
Male	59	Reference			
Female	33	0.757 (0.456–1.255)	0.28		
Expression level of SP100	92	1.753 (1.177–2.612)	**0.006**	1.357 (0.632–2.914)	0.02
Jaundice	92		0.808		
No	67	Reference			
Mild jaundice	4	1.312 (0.468–3.680)	0.606		
Moderate jaundice	19	0.794 (0.439–1.438)	0.447		
Severe jaundice	2	1.189 (0.163–8.679)	0.865		
Drinking Head history	92		0.546		
No	67	Reference			
Yes	25	0.853 (0.506–1.439)	0.551		
History of diabetes	92		0.103		
No	70	Reference			
Yes	22	1.575 (0.931–2.664)	0.09		
Smoking history	92		**0.012**		
No	70	Reference		Reference	
Yes	22	2.197 (1.232–3.916)	**0.008**	1.421 (0.524–3.855)	0.491
Size (cm)	92	1.017 (0.886–1.166)	0.815		
Site	92		0.977		
Head	53	Reference			
Body	9	1.054 (0.471–2.356)	0.899		
Body and tail	30	1.051 (0.639–1.731)	0.843		
TNM staging	92		**0.048**		
1A	1	Reference		Reference	
1B	16	0.093 (0.011–0.782)	**0.029**	0.071 (0.008–0.607)	0.016
2A	26	0.187 (0.024–1.456)	0.109	0.107 (0.013–0.890)	0.039
2B	34	0.272 (0.036–2.073)	0.209	0.113 (0.012–1.029)	0.053
3A	7	0.280 (0.032–2.424)	0.248	0.141 (0.015–1.336)	0.088
3B	4	0.372 (0.040–3.435)	0.383	0.142 (0.013–1.585)	0.113
4A	4	0.429 (0.046–3.966)	0.456	0.169 (0.016–1.827)	0.143
N staging	92		0.051		
N0	52	Reference		Reference	
N1	40	1.606 (1.001–2.577)	**0.049**	1.390 (0.670–2.883)	0.376
Pathological grade	92		0.543		
G1	14	Reference			
G2	58	0.810 (0.432–1.518)	0.511		
G3	20	1.098 (0.532–2.268)	0.800		
Nerve invasion	92		**0.015**		
No	38	Reference		Reference	
Yes	54	1.808 (1.111–2.943)	**0.017**	1.588 (0.934–2.701)	0.088
Macrovascular invasion	92		**0.014**		
No	61	Reference		Reference	
Yes	31	1.929 (1.158–3.214)	**0.012**	0.852 (0.314–2.312)	0.754
CA199 (U/mL)	92	1.000 (1.000–1.001)	0.255		

## DISCUSSION

The SP100 family members encompass HSR and SAND domains, which are pivotal in transcription, cell division, senescence, and response to DNA damage or infection [3–5]. Consequently, a notable elevation in the expression levels of SP100 family members can exert a substantial influence on cellular fate. Moreover, the precise role of SP100 family members in PAAD remains elusive, with limited available studies on the subject. This study represents a pioneering endeavor, incorporating bioinformatics analyses to elucidate the involvement of SP100 family members in PAAD comprehensively. It unveils their functions in PAAD and proposes plausible mechanisms by which SP100 members contribute to the initiation and progression of PAAD, utilizing diverse analytical approaches such as gene expression analysis, genetic variation assessment, promoter methylation examination, evaluation of immune cell infiltration, gene enrichment analysis, protein-protein interaction network exploration, and investigation of drug sensitivity.

Mutations in the tumor suppressor gene TP53 have been implicated in the development of various malignant tumors, including PAAD, esophageal cancer, and lung cancer [21–23]. In our investigation of the underlying mechanisms, we made an intriguing discovery: the expression levels of SP100 family members were significantly higher in PAAD tissues with TP53 mutations compared to those with wild-type TP53. This observation suggests that SP100 family members may serve as downstream targets influenced by mutant TP53. Moreover, our constructed protein-protein interaction network highlighted the strong influence of TP53 on the SP100 family members, emphasizing the close connection between TP53 and the SP100 family members. Given the significant correlation between high expression of SP100 family members in PAAD and the occurrence, progression, and unfavorable prognosis of patients, it is plausible to propose that the association between TP53 mutation and the expression level of SP100 family members may underlie the role of SP100 family members in PAAD development. Another noteworthy finding was the coexpression of SP100 family members with M6A methylation regulators. Numerous studies have identified M6A methylation regulators as cancer-promoting factors, and elevated expression of these regulators has been closely linked to the initiation and progression of various malignancies, including colorectal cancer, PAAD, and hepatocellular carcinoma [24–33]. The heightened expression of SP100 family members may contribute to increased expression of M6A methylation regulators, consequently amplifying the carcinogenic effects of these regulators in PAAD.

To gain further insights into the potential carcinogenic mechanism of the SP100 family members in PAAD, we conducted a heatmap analysis of 200 genes associated with the SP100 family, utilizing GeneCards database (https://www.genecards.org/), without annotation. Notably, we identified 14 highly carcinogenic genes, including 13 protein-coding genes such as PARP14, EPSTI1, NMI, and the long non-coding RNA HCP5. These findings highlight the intricate interplay between the SP100 family members and key genes implicated in cancer development. Moreover, our investigation revealed that the SP100 family members play crucial activating roles in the epithelial-mesenchymal transition (EMT) pathway and hormone/ER pathways. Additionally, SP140L was found to activate the PI3K/AKT and RAS/MAPK pathways. The EMT signaling pathway, a well-known driver of tumorigenesis, has been implicated in various malignancies, including PAAD, prostate cancer, and breast cancer [34, 35]. Activation of this pathway enhances the mobility and invasiveness of tumor cells, thereby facilitating the formation of secondary metastatic lesions [36, 37]. Furthermore, the PI3K/AKT and RAS/MAPK signaling pathways are recognized for their role in promoting the growth and proliferation of tumor cells [19, 20]. The coexpression of SP100 family members with multiple oncogenic genes and their enrichment in various oncogenic pathways provide additional insights into the underlying mechanisms by which the SP100 family members contribute to the initiation and progression of PAAD. These findings shed light on the intricate molecular networks and potential therapeutic targets involving the SP100 family members in PAAD.

Finally, we analyzed the potential value of SP100 family members in the clinical treatment of PAAD. The results showed significant coexpression of SP100 family members with multiple immune checkpoint genes, including CD44, HHLA2 and HAVCR2. Some immune checkpoint genes play a role in promoting cancer in malignant tumors, such as in PAAD, lymphoma and malignant pleural mesothelioma (https://www.genecards.org/), so the research and development of immune checkpoint inhibitors targeting these genes is of great significance for the treatment of PAAD. In addition, we found that the expression levels of SP140 and SP140L were positively correlated with sensitivity to 5-fluorouracil and paclitaxel, respectively. Therefore, PAAD patients with high SP140 and SP140L expression may respond better to 5-fluorouracil and paclitaxel.

This pioneering study represents the first comprehensive bioinformatics analysis investigating the functional significance of SP100 family members in PAAD. The findings reveal a compelling association between elevated expression of SP100 family members and adverse clinicopathological features as well as poor prognosis in PAAD patients. Notably, our study unraveled a remarkable diagnostic potential of SP100 family members, with superior area under the curve (AUC) values in distinguishing normal pancreatic tissues from PAAD tissues. In fact, our minimum AUC value of 0.913 surpassed the maximum AUC values reported in previous studies examining biomarkers for PAAD diagnosis and prognosis [38, 39]. This underscores the exceptional discriminatory and calibration characteristics offered by the SP100 family. Furthermore, our investigations encompassed a comprehensive literature review on PAAD diagnosis and prognosis, leading to the identification of intriguing associations. We discovered the coexpression of SP100 family members with M6A methylation regulators and various oncogenic genes. Moreover, the enrichment of SP100 family members in diverse carcinogenic pathways and their close relationship with TP53 mutation status suggest potential mechanisms through which SP100 family members contribute to the initiation and progression of PAAD. The coexpression of SP100 family members with multiple cancer-promoting immune checkpoint genes and their positive correlation with sensitivity to specific chemotherapeutic agents, such as 5-fluorouracil and paclitaxel, provide valuable insights for clinical translation of our findings. Despite these significant contributions, this study has some limitations that warrant consideration. Firstly, the inclusion of a limited number of datasets may restrict the generalizability of the findings. Additionally, the current study primarily relies on bioinformatics analyses to elucidate the functions and mechanisms of SP100 family members in PAAD. Therefore, there is an urgent need for experimental studies to validate the cancer-promoting effects of SP100 family members in PAAD and further elucidate the underlying mechanisms. Overall, this study unveils the pivotal role of SP100 family members in PAAD and sets the stage for future investigations that integrate experimental approaches to confirm their oncogenic potential. The clinical implications derived from our findings offer promising avenues for the development of diagnostic and therapeutic strategies targeting the SP100 family in PAAD.

## CONCLUSION

In summary, our comprehensive bioinformatics analysis of the SP100 family in PAAD sheds light on their crucial involvement in the onset and progression of this disease, thereby highlighting their potential as promising therapeutic targets and prognostic biomarkers for PAAD patients. While our findings are compelling, it is essential to validate them through rigorous multicenter studies with large sample sizes in the future. Moreover, there is a pressing need for functional experiments and mechanistic investigations to further elucidate the underlying molecular mechanisms. By pursuing these avenues, we can enhance our understanding of the SP100 family’s role in PAAD and pave the way for the development of innovative therapeutic strategies and improved patient outcomes.

## Supplementary Materials

Supplementary Figures

Supplementary Table 1

Supplementary Table 2

Supplementary Table 3

Supplementary Table 4

Supplementary Table 5

Supplementary Table 6

Supplementary Table 7

Supplementary Table 8

Supplementary Table 9

Supplementary Table 10

Supplementary Table 11
