# Supplementary material for: Expression, prognostic value and mechanism of SP100 family in pancreatic adenocarcinoma
Source: Aging (Albany NY). 2023 Jun 22;15(12):5569–91. doi: 10.18632/aging.204811 (PMC10333092; doi:10.18632/aging.204811)
Supplement: Supplementary Figures [file aging-15-204811-s001.pdf]

SUPPLEMENTARY FIGURES

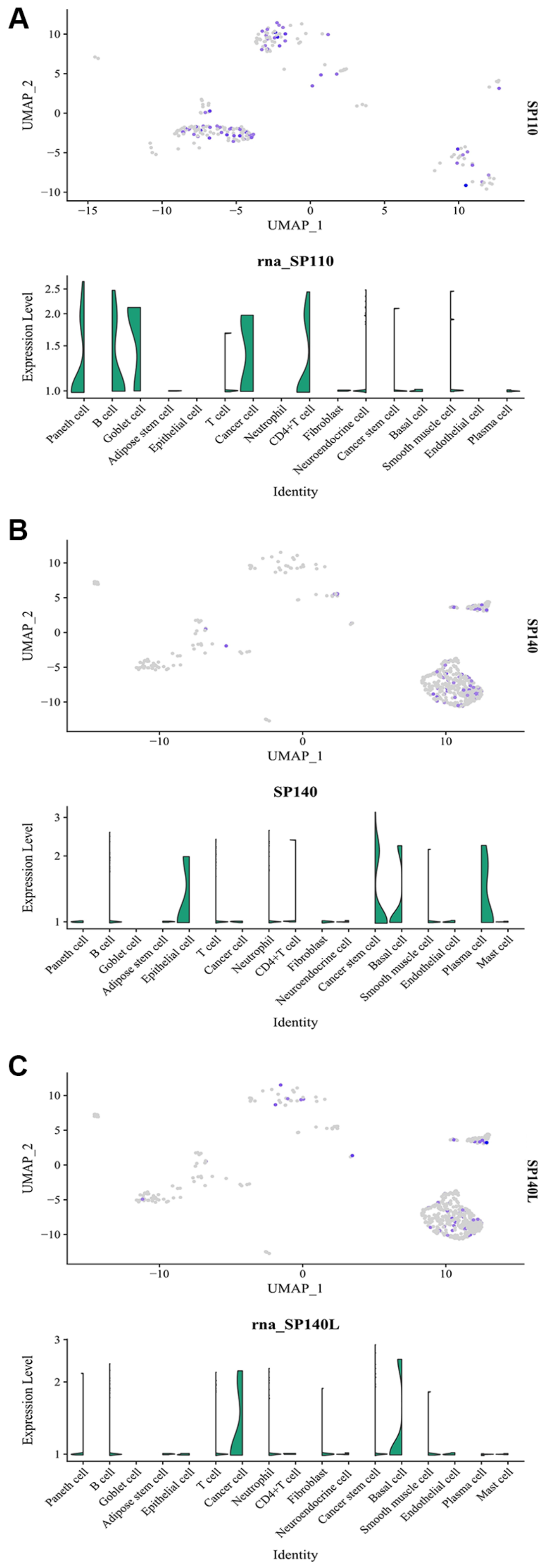

Supplementary Figure 1. Expression characteristics of SP110/SP140/SP140L at the single-cell level in PAAD. (**A**) The single-cell level of SP110; (**B**) The single-cell level of SP140; (**C**) The single-cell level of SP140L.

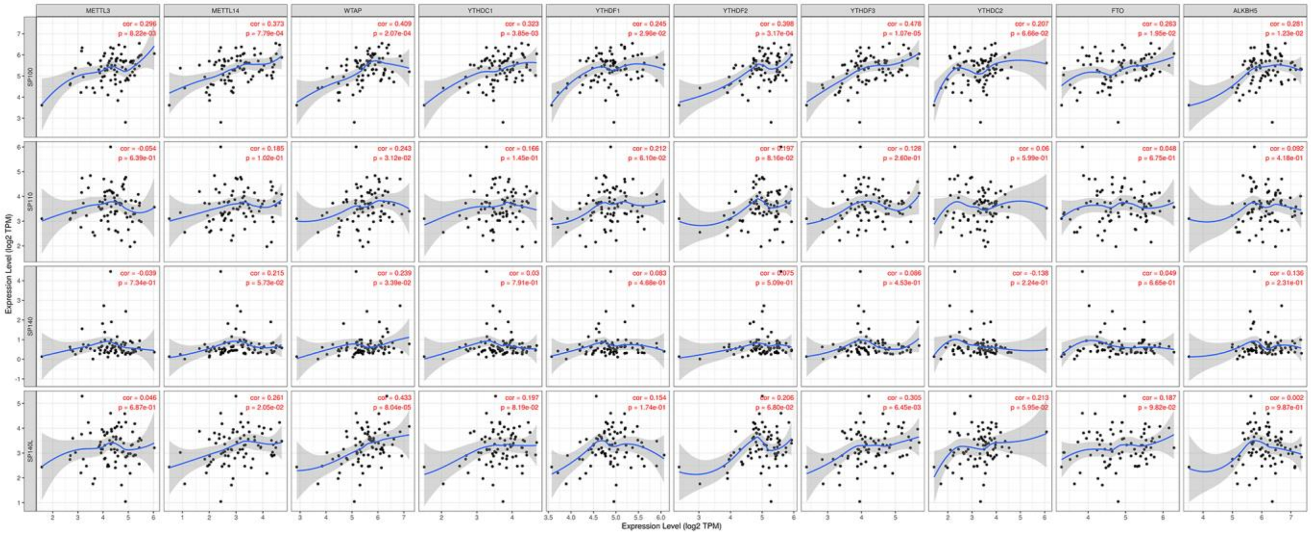

Supplementary Figure 2. SP100 family members were positively correlated with the expression levels of most M6A methylation regulators.

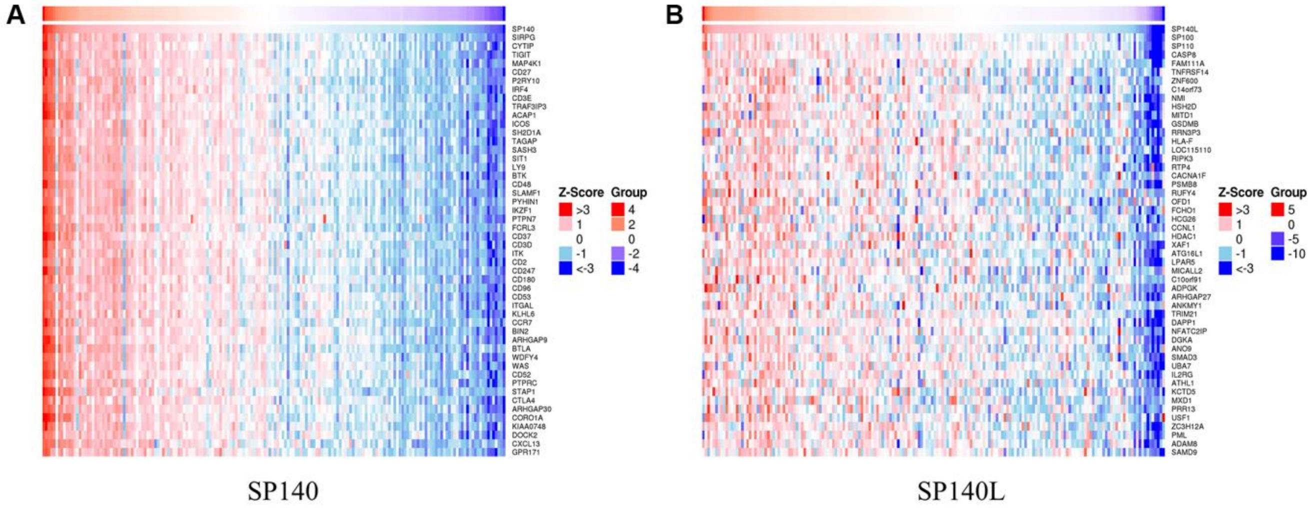

Supplementary Figure 3. Top 50 genes co-expressed with SP140/SP140L. (**A**) Top 50 genes co-expressed with SP140; (**B**) Top 50 genes co-expressed with SP140L.

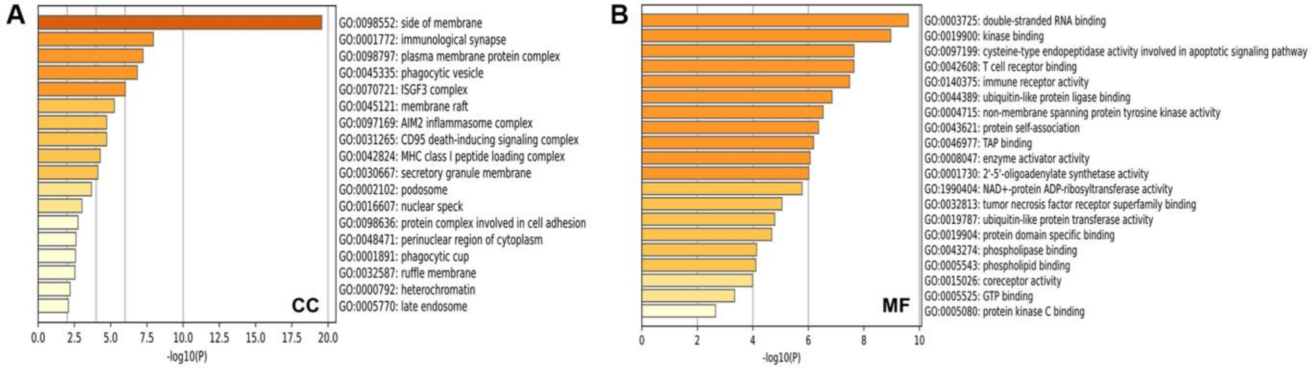

Supplementary Figure 4. The GO enrichment of the CC terms and MF terms of the SP100 family and its 400 co-expressed genes. (**A**) CC terms; (**B**) MF terms.
