# Supplementary material for: Expression, prognostic value and mechanism of SP100 family in pancreatic adenocarcinoma
Source: Aging (Albany NY). 2023 Jun 22;15(12):5569–91. doi: 10.18632/aging.204811 (PMC10333092; doi:10.18632/aging.204811)
Supplement: Supplementary Table 2 [file aging-15-204811-s003.docx]

| Characteristic  **Supplementary Table 2. Relationship between the SP100 family expression and clinicopathological characteristics of PAAD patients.** | Low expression of SP100 | High expression of SP100 | p |
| --- | --- | --- | --- |
| n | 89 | 89 |  |
| T1 | 7 (4%) | 0 (0%) |  |
| T2 | 15 (8.5%) | 9 (5.1%) |  |
| T3 | 62 (35.2%) | 80 (45.5%) |  |
| T4 | 3 (1.7%) | 0 (0%) |  |
| N stage, n (%) |  |  | 0.026 |
| N0 | 32 (18.5%) | 18 (10.4%) |  |
| N1 | 54 (31.2%) | 69 (39.9%) |  |
| M stage, n (%) |  |  | 1.000 |
| M0 | 33 (39.3%) | 46 (54.8%) |  |
| M1 | 2 (2.4%) | 3 (3.6%) |  |
| Pathologic stage, n (%) |  |  | < 0.001 |
| Stage I | 18 (10.3%) | 3 (1.7%) |  |
| Stage II | 64 (36.6%) | 82 (46.9%) |  |
| Stage III | 3 (1.7%) | 0 (0%) |  |
| Stage IV | 2 (1.1%) | 3 (1.7%) |  |
| Radiation therapy, n (%) |  |  | 0.717 |
| No | 60 (36.8%) | 58 (35.6%) |  |
| Yes | 25 (15.3%) | 20 (12.3%) |  |
| Primary therapy outcome, n (%) |  |  | 0.321 |
| PD | 22 (15.8%) | 27 (19.4%) |  |
| SD | 7 (5%) | 2 (1.4%) |  |
| PR | 6 (4.3%) | 4 (2.9%) |  |
| CR | 36 (25.9%) | 35 (25.2%) |  |
| Gender, n (%) |  |  | 0.175 |
| Female | 45 (25.3%) | 35 (19.7%) |  |
| Male | 44 (24.7%) | 54 (30.3%) |  |
| Race, n (%) |  |  | 0.852 |
| Asian | 5 (2.9%) | 6 (3.4%) |  |
| Black or African American | 4 (2.3%) | 2 (1.1%) |  |
| White | 78 (44.8%) | 79 (45.4%) |  |
| Age, n (%) |  |  | 0.230 |
| <=65 | 42 (23.6%) | 51 (28.7%) |  |
| >65 | 47 (26.4%) | 38 (21.3%) |  |
| Residual tumor, n (%) |  |  | 0.591 |
| R0 | 58 (35.4%) | 49 (29.9%) |  |
| R1 | 24 (14.6%) | 28 (17.1%) |  |
| R2 | 2 (1.2%) | 3 (1.8%) |  |
| Anatomic neoplasm subdivision, n (%) |  |  | 0.590 |
| Head of Pancreas | 71 (39.9%) | 67 (37.6%) |  |
| Other | 18 (10.1%) | 22 (12.4%) |  |
| Histologic grade, n (%) |  |  | < 0.001 |
| G1 | 24 (13.6%) | 7 (4%) |  |
| G2 | 46 (26.1%) | 49 (27.8%) |  |
| G3 | 15 (8.5%) | 33 (18.8%) |  |
| G4 | 2 (1.1%) | 0 (0%) |  |
| Smoker, n (%) |  |  | 0.375 |
| No | 37 (25.7%) | 28 (19.4%) |  |
| Yes | 38 (26.4%) | 41 (28.5%) |  |
| Alcohol history, n (%) |  |  | 0.312 |
| No | 30 (18.1%) | 35 (21.1%) |  |
| Yes | 56 (33.7%) | 45 (27.1%) |  |
| History of diabetes, n (%) |  |  | 0.337 |
| No | 60 (41.1%) | 48 (32.9%) |  |
| Yes | 17 (11.6%) | 21 (14.4%) |  |
| History of chronic pancreatitis, n (%) |  |  | 0.693 |
| No | 66 (46.8%) | 62 (44%) |  |
| Yes | 8 (5.7%) | 5 (3.5%) |  |
| Family history of cancer, n (%) |  |  | 0.408 |
| No | 23 (20.9%) | 24 (21.8%) |  |
| Yes | 37 (33.6%) | 26 (23.6%) |  |
| Age, mean ± SD | 65.89 ± 11.22 | 63.61 ± 10.29 | 0.159 |

| Characteristic | Low expression of SP110 | High expression of SP110 | p |
| --- | --- | --- | --- |
| n | 89 | 89 |  |
| T stage, n (%) |  |  | 0.164 |
| T1 | 5 (2.8%) | 2 (1.1%) |  |
| T2 | 13 (7.4%) | 11 (6.2%) |  |
| T3 | 66 (37.5%) | 76 (43.2%) |  |
| T4 | 3 (1.7%) | 0 (0%) |  |
| N stage, n (%) |  |  | 0.026 |
| N0 | 32 (18.5%) | 18 (10.4%) |  |
| N1 | 54 (31.2%) | 69 (39.9%) |  |
| M stage, n (%) |  |  | 0.155 |
| M0 | 31 (36.9%) | 48 (57.1%) |  |
| M1 | 4 (4.8%) | 1 (1.2%) |  |
| Pathologic stage, n (%) |  |  | 0.002 |
| Stage I | 16 (9.1%) | 5 (2.9%) |  |
| Stage II | 64 (36.6%) | 82 (46.9%) |  |
| Stage III | 3 (1.7%) | 0 (0%) |  |
| Stage IV | 4 (2.3%) | 1 (0.6%) |  |
| Radiation therapy, n (%) |  |  | 0.885 |
| No | 61 (37.4%) | 57 (35%) |  |
| Yes | 22 (13.5%) | 23 (14.1%) |  |
| Primary therapy outcome, n (%) |  |  | 0.205 |
| PD | 19 (13.7%) | 30 (21.6%) |  |
| SD | 6 (4.3%) | 3 (2.2%) |  |
| PR | 6 (4.3%) | 4 (2.9%) |  |
| CR | 39 (28.1%) | 32 (23%) |  |
| Gender, n (%) |  |  | 0.451 |
| Female | 43 (24.2%) | 37 (20.8%) |  |
| Male | 46 (25.8%) | 52 (29.2%) |  |
| Race, n (%) |  |  | 0.741 |
| Asian | 5 (2.9%) | 6 (3.4%) |  |
| Black or African American | 2 (1.1%) | 4 (2.3%) |  |
| White | 80 (46%) | 77 (44.3%) |  |
| Age, n (%) |  |  | 0.368 |
| <=65 | 43 (24.2%) | 50 (28.1%) |  |
| >65 | 46 (25.8%) | 39 (21.9%) |  |
| Residual tumor, n (%) |  |  | 0.416 |
| R0 | 56 (34.1%) | 51 (31.1%) |  |
| R1 | 26 (15.9%) | 26 (15.9%) |  |
| R2 | 1 (0.6%) | 4 (2.4%) |  |
| Histologic grade, n (%) |  |  | < 0.001 |
| G1 | 22 (12.5%) | 9 (5.1%) |  |
| G2 | 49 (27.8%) | 46 (26.1%) |  |
| G3 | 14 (8%) | 34 (19.3%) |  |
| G4 | 2 (1.1%) | 0 (0%) |  |
| Anatomic neoplasm subdivision, n (%) |  |  | 1.000 |
| Head of Pancreas | 69 (38.8%) | 69 (38.8%) |  |
| Other | 20 (11.2%) | 20 (11.2%) |  |
| Smoker, n (%) |  |  | 0.222 |
| No | 38 (26.4%) | 27 (18.8%) |  |
| Yes | 37 (25.7%) | 42 (29.2%) |  |
| Alcohol history, n (%) |  |  | 1.000 |
| No | 33 (19.9%) | 32 (19.3%) |  |
| Yes | 51 (30.7%) | 50 (30.1%) |  |
| History of diabetes, n (%) |  |  | 0.156 |
| No | 59 (40.4%) | 49 (33.6%) |  |
| Yes | 15 (10.3%) | 23 (15.8%) |  |
| History of chronic pancreatitis, n (%) |  |  | 1.000 |
| No | 64 (45.4%) | 64 (45.4%) |  |
| Yes | 7 (5%) | 6 (4.3%) |  |
| Family history of cancer, n (%) |  |  | 0.582 |
| No | 22 (20%) | 25 (22.7%) |  |
| Yes | 34 (30.9%) | 29 (26.4%) |  |
| Age, mean ± SD | 65.76 ± 10.35 | 63.73 ± 11.2 | 0.210 |

| Characteristic | Low expression of SP140 | High expression of SP140 | p |
| --- | --- | --- | --- |
| n | 89 | 89 |  |
| T stage, n (%) |  |  | 0.591 |
| T1 | 5 (2.8%) | 2 (1.1%) |  |
| T2 | 13 (7.4%) | 11 (6.2%) |  |
| T3 | 68 (38.6%) | 74 (42%) |  |
| T4 | 1 (0.6%) | 2 (1.1%) |  |
| N stage, n (%) |  |  | 0.187 |
| N0 | 29 (16.8%) | 21 (12.1%) |  |
| N1 | 56 (32.4%) | 67 (38.7%) |  |
| M stage, n (%) |  |  | 0.164 |
| M0 | 33 (39.3%) | 46 (54.8%) |  |
| M1 | 4 (4.8%) | 1 (1.2%) |  |
| Pathologic stage, n (%) |  |  | 0.142 |
| Stage I | 14 (8%) | 7 (4%) |  |
| Stage II | 67 (38.3%) | 79 (45.1%) |  |
| Stage III | 1 (0.6%) | 2 (1.1%) |  |
| Stage IV | 4 (2.3%) | 1 (0.6%) |  |
| Radiation therapy, n (%) |  |  | 1.000 |
| No | 60 (36.8%) | 58 (35.6%) |  |
| Yes | 23 (14.1%) | 22 (13.5%) |  |
| Primary therapy outcome, n (%) |  |  | 0.808 |
| PD | 22 (15.8%) | 27 (19.4%) |  |
| SD | 5 (3.6%) | 4 (2.9%) |  |
| PR | 6 (4.3%) | 4 (2.9%) |  |
| CR | 33 (23.7%) | 38 (27.3%) |  |
| Gender, n (%) |  |  | 0.651 |
| Female | 38 (21.3%) | 42 (23.6%) |  |
| Male | 51 (28.7%) | 47 (26.4%) |  |
| Race, n (%) |  |  | 0.112 |
| Asian | 8 (4.6%) | 3 (1.7%) |  |
| Black or African American | 1 (0.6%) | 5 (2.9%) |  |
| White | 78 (44.8%) | 79 (45.4%) |  |
| Age, n (%) |  |  | 0.133 |
| <=65 | 41 (23%) | 52 (29.2%) |  |
| >65 | 48 (27%) | 37 (20.8%) |  |
| Residual tumor, n (%) |  |  | 0.916 |
| R0 | 53 (32.3%) | 54 (32.9%) |  |
| R1 | 25 (15.2%) | 27 (16.5%) |  |
| R2 | 3 (1.8%) | 2 (1.2%) |  |
| Histologic grade, n (%) |  |  | 0.099 |
| G1 | 21 (11.9%) | 10 (5.7%) |  |
| G2 | 45 (25.6%) | 50 (28.4%) |  |
| G3 | 20 (11.4%) | 28 (15.9%) |  |
| G4 | 1 (0.6%) | 1 (0.6%) |  |
| Anatomic neoplasm subdivision, n (%) |  |  | < 0.001 |
| Head of Pancreas | 59 (33.1%) | 79 (44.4%) |  |
| Other | 30 (16.9%) | 10 (5.6%) |  |
| Smoker, n (%) |  |  | 0.024 |
| No | 42 (29.2%) | 23 (16%) |  |
| Yes | 35 (24.3%) | 44 (30.6%) |  |
| Alcohol history, n (%) |  |  | 0.709 |
| No | 32 (19.3%) | 33 (19.9%) |  |
| Yes | 54 (32.5%) | 47 (28.3%) |  |
| History of diabetes, n (%) |  |  | 0.337 |
| No | 60 (41.1%) | 48 (32.9%) |  |
| Yes | 17 (11.6%) | 21 (14.4%) |  |
| History of chronic pancreatitis, n (%) |  |  | 0.159 |
| No | 71 (50.4%) | 57 (40.4%) |  |
| Yes | 4 (2.8%) | 9 (6.4%) |  |
| Family history of cancer, n (%) |  |  | 1.000 |
| No | 24 (21.8%) | 23 (20.9%) |  |
| Yes | 32 (29.1%) | 31 (28.2%) |  |
| Age, median (IQR) | 66 (61, 74) | 64 (56, 72) | 0.086 |

| Characteristic | Low expression of SP140L | High expression of SP140L | p |
| --- | --- | --- | --- |
| n | 89 | 89 |  |
| T stage, n (%) |  |  | 0.064 |
| T1 | 5 (2.8%) | 2 (1.1%) |  |
| T2 | 15 (8.5%) | 9 (5.1%) |  |
| T3 | 64 (36.4%) | 78 (44.3%) |  |
| T4 | 3 (1.7%) | 0 (0%) |  |
| N stage, n (%) |  |  | 0.026 |
| N0 | 32 (18.5%) | 18 (10.4%) |  |
| N1 | 54 (31.2%) | 69 (39.9%) |  |
| M stage, n (%) |  |  | 0.665 |
| M0 | 37 (44%) | 42 (50%) |  |
| M1 | 3 (3.6%) | 2 (2.4%) |  |
| Pathologic stage, n (%) |  |  | 0.006 |
| Stage I | 16 (9.1%) | 5 (2.9%) |  |
| Stage II | 65 (37.1%) | 81 (46.3%) |  |
| Stage III | 3 (1.7%) | 0 (0%) |  |
| Stage IV | 3 (1.7%) | 2 (1.1%) |  |
| Radiation therapy, n (%) |  |  | 0.809 |
| No | 62 (38%) | 56 (34.4%) |  |
| Yes | 22 (13.5%) | 23 (14.1%) |  |
| Primary therapy outcome, n (%) |  |  | 0.288 |
| PD | 19 (13.7%) | 30 (21.6%) |  |
| SD | 6 (4.3%) | 3 (2.2%) |  |
| PR | 5 (3.6%) | 5 (3.6%) |  |
| CR | 38 (27.3%) | 33 (23.7%) |  |
| Gender, n (%) |  |  | 0.880 |
| Female | 41 (23%) | 39 (21.9%) |  |
| Male | 48 (27%) | 50 (28.1%) |  |
| Race, n (%) |  |  | 0.112 |
| Asian | 3 (1.7%) | 8 (4.6%) |  |
| Black or African American | 5 (2.9%) | 1 (0.6%) |  |
| White | 79 (45.4%) | 78 (44.8%) |  |
| Residual tumor, n (%) |  |  | 0.163 |
| R0 | 61 (37.2%) | 46 (28%) |  |
| R1 | 22 (13.4%) | 30 (18.3%) |  |
| R2 | 2 (1.2%) | 3 (1.8%) |  |
| Histologic grade, n (%) |  |  | 0.122 |
| G1 | 20 (11.4%) | 11 (6.2%) |  |
| G2 | 45 (25.6%) | 50 (28.4%) |  |
| G3 | 21 (11.9%) | 27 (15.3%) |  |
| G4 | 2 (1.1%) | 0 (0%) |  |
| Smoker, n (%) |  |  | 0.880 |
| No | 33 (22.9%) | 32 (22.2%) |  |
| Yes | 38 (26.4%) | 41 (28.5%) |  |
| Anatomic neoplasm subdivision, n (%) |  |  | 0.590 |
| Head of Pancreas | 67 (37.6%) | 71 (39.9%) |  |
| Other | 22 (12.4%) | 18 (10.1%) |  |
| Alcohol history, n (%) |  |  | 1.000 |
| No | 33 (19.9%) | 32 (19.3%) |  |
| Yes | 52 (31.3%) | 49 (29.5%) |  |
| History of diabetes, n (%) |  |  | 0.446 |
| No | 50 (34.2%) | 58 (39.7%) |  |
| Yes | 21 (14.4%) | 17 (11.6%) |  |
| History of chronic pancreatitis, n (%) |  |  | 1.000 |
| No | 64 (45.4%) | 64 (45.4%) |  |
| Yes | 7 (5%) | 6 (4.3%) |  |
| Family history of cancer, n (%) |  |  | 0.378 |
| No | 22 (20%) | 25 (22.7%) |  |
| Yes | 36 (32.7%) | 27 (24.5%) |  |
| Age, mean ± SD | 66.49 ± 10.02 | 63 ± 11.31 | 0.030 |
