# Supplementary material for: Expression, prognostic value and mechanism of SP100 family in pancreatic adenocarcinoma
Source: Aging (Albany NY). 2023 Jun 22;15(12):5569–91. doi: 10.18632/aging.204811 (PMC10333092; doi:10.18632/aging.204811)
Supplement: Supplementary Table 6 [file aging-15-204811-s007.docx]

**Supplementary Table 6. Enrichment analysis of the SP100 family and its 400 co-expressed genes.**

ExpandCategory TermID Description LogP

GO Biological Processes GO:0009615 response to virus -50.73957

GO Biological Processes GO:0050778 positive regulation of immune response -40.44458

GO Biological Processes GO:0045321 leukocyte activation -36.52153

GO Biological Processes GO:0050865 regulation of cell activation -35.04258

GO Biological Processes GO:0031347 regulation of defense response -34.56356

GO Biological Processes GO:0001819 positive regulation of cytokine production -31.69747

GO Biological Processes GO:0002683 negative regulation of immune system process -30.49944

GO Biological Processes GO:0071345 cellular response to cytokine stimulus -25.17348

GO Biological Processes GO:0002697 regulation of immune effector process -21.50133

GO Biological Processes GO:0009617 response to bacterium -15.25237

GO Biological Processes GO:0045058 T cell selection -14.20212

GO Biological Processes GO:0043122 regulation of I-kappaB kinase/NF-kappaB signaling -13.81122

GO Biological Processes GO:0002252 immune effector process -13.33527

GO Biological Processes GO:0002695 negative regulation of leukocyte activation -13.03419

GO Biological Processes GO:0035456 response to interferon-beta -12.36810

GO Biological Processes GO:0050854 regulation of antigen receptor-mediated signaling pathway -11.64512

GO Biological Processes GO:0034341 response to interferon-gamma -11.61215

GO Biological Processes GO:0035455 response to interferon-alpha -11.0088

GO Biological Processes GO:0032649 regulation of interferon-gamma production -10.90714

GO Biological Processes GO:0002221 pattern recognition receptor signaling pathway -10.80513

KEGG Pathway hsa05169 Epstein-Barr virus infection -23.08828

KEGG Pathway hsa05340 Primary immunodeficiency -16.51313

KEGG Pathway hsa04621 NOD-like receptor signaling pathway -13.41319

KEGG Pathway hsa04514 Cell adhesion molecules -12.77617

KEGG Pathway hsa04660 T cell receptor signaling pathway -11.61914

KEGG Pathway hsa04659 Th17 cell differentiation -11.39014

KEGG Pathway hsa05170 Human immunodeficiency virus 1 infection -11.27918

KEGG Pathway hsa04060 Cytokine-cytokine receptor interaction -9.78619

KEGG Pathway hsa05166 Human T-cell leukemia virus 1 infection -8.12615

KEGG Pathway hsa04640 Hematopoietic cell lineage -7.24010

KEGG Pathway hsa04062 Chemokine signaling pathway -7.13113

KEGG Pathway hsa05161 Hepatitis B -7.05312

KEGG Pathway hsa04662 B cell receptor signaling pathway -5.7888

KEGG Pathway hsa04650 Natural killer cell mediated cytotoxicity -5.1649

KEGG Pathway hsa04064 NF-kappa B signaling pathway -5.0118

KEGG Pathway hsa04668 TNF signaling pathway -4.7748

KEGG Pathway hsa04623 Cytosolic DNA-sensing pathway -4.4136

KEGG Pathway hsa03250 Viral life cycle – HIV -1-4.4136

KEGG Pathway hsa04622 RIG-I-like receptor signaling pathway -4.1536

KEGG Pathway hsa05135 Yersinia infection -4.1478

GO Cellular Components GO:0098552 side of membrane -19.56341

GO Cellular Components GO:0001772 immunological synapse -7.9508

GO Cellular Components GO:0098797 plasma membrane protein complex -7.23025

GO Cellular Components GO:0045335 phagocytic vesicle -6.83611

GO Cellular Components GO:0070721 ISGF3 complex -6.0133

GO Cellular Components GO:0045121 membrane raft -5.25214

GO Cellular Components GO:0097169 AIM2 inflammasome complex -4.7213

GO Cellular Components GO:0031265 CD95 death-inducing signaling complex -4.7213

GO Cellular Components GO:0042824 MHC class I peptide loading complex -4.2813

GO Cellular Components GO:0030667 secretory granule membrane -4.12112

GO Cellular Components GO:0002102 podosome -3.6714

GO Cellular Components GO:0016607 nuclear speck -3.02012

GO Cellular Components GO:0098636 protein complex involved in cell adhesion -2.7504

GO Cellular Components GO:0048471 perinuclear region of cytoplasm -2.60216

GO Cellular Components GO:0001891 phagocytic cup -2.5773

GO Cellular Components GO:0032587 ruffle membrane -2.5435

GO Cellular Components GO:0000792 heterochromatin -2.1964

GO Cellular Components GO:0005770 late endosome -2.0818

GO Molecular Functions GO:0003725 double-stranded RNA binding -9.60211

GO Molecular Functions GO:0019900 kinase binding -8.97029

GO Molecular Functions GO:0042608 T cell receptor binding -7.6455

GO Molecular Functions GO:0097199cysteine-type endopeptidase activity involved in apoptotic signaling pathway -7.6455

GO Molecular Functions GO:0140375 immune receptor activity -7.48812

GO Molecular Functions GO:0044389 ubiquitin-like protein ligase binding -6.85416

GO Molecular Functions GO:0004715 non-membrane spanning protein tyrosine kinase activity -6.5347

GO Molecular Functions GO:0043621 protein self-association -6.3698

GO Molecular Functions GO:0046977 TAP binding -6.1894

GO Molecular Functions GO:0008047 enzyme activator activity -6.06520

GO Molecular Functions GO:0001730 2'-5'-oligoadenylate synthetase activity -6.0133

GO Molecular Functions GO:1990404 NAD+-protein ADP-ribosyltransferase activity -5.7775

GO Molecular Functions GO:0032813 tumor necrosis factor receptor superfamily binding -5.0496

GO Molecular Functions GO:0019787 ubiquitin-like protein transferase activity -4.78816

GO Molecular Functions GO:0019904 protein domain specific binding -4.68120

GO Molecular Functions GO:0043274 phospholipase binding -4.1384

GO Molecular Functions GO:0005543 phospholipid binding -4.11315

GO Molecular Functions GO:0015026 coreceptor activity -3.9915

GO Molecular Functions GO:0005525 GTP binding -3.34512

GO Molecular Functions GO:0005080 protein kinase C binding -2.6604
