# Supplementary material for: Expression, prognostic value and mechanism of SP100 family in pancreatic adenocarcinoma
Source: Aging (Albany NY). 2023 Jun 22;15(12):5569–91. doi: 10.18632/aging.204811 (PMC10333092; doi:10.18632/aging.204811)
Supplement: Supplementary Table 8 [file aging-15-204811-s009.pdf]

Supplementary Table 8. Functional intensity scoring of 24 interacting genes.

Gene	Degree	Number of undirected edges	Topological Coefficient	Radiality
TP53	17	17	0.419786096	0.98209719
UBE2I	16	16	0.413043478	0.98209719
SUMO1	15	15	0.434782609	0.97953964
SP100	14	14	0.444805195	0.9769821
SUMO2	14	14	0.450310559	0.9769821
PIAS1	11	11	0.508264463	0.96675192
HSPA4	11	11	0.450413223	0.96675192
PML	11	11	0.505928854	0.96930946
DAXX	10	10	0.519047619	0.96419437
RANBP2	10	10	0.559090909	0.96419437
STUB1	8	8	0.428571429	0.95907928
NUP210	8	8	0.541666667	0.95907928
UBE3A	7	7	0.571428571	0.95396419
DNAJB1	6	6	0.5	0.94884911
SIRT1	6	6	0.625	0.94884911
UBA2	6	6	0.715686275	0.94117647
AURKA	5	5	0.673684211	0.94373402
RPA1	5	5	0.705263158	0.94373402
DDX5	5	5	0.768421053	0.94373402
CLPP	4	4	0.573529412	0.93606138
H3F3B	3	3	0.650793651	0.94373402
SP110	2	2	0.714285714	0.92838875
SP140L	1	1	0	0.90792839
SP140	1	1	0	0.9028133
